# A Systematic Review and Meta-Analysis on the Efficacy and Safety of Concomitant Laparoscopic Cholecystectomy and Sleeve Gastrectomy in Patients with Morbid Obesity

**DOI:** 10.3390/jcm14124108

**Published:** 2025-06-10

**Authors:** Walid M. Abd El Maksoud, Khaled S. Abbas, Fahad S. Al Amri, Hassan A. Alzahrani, Abdullah Dalboh, Marei H. Alshandeer, Maha A. Alghamdi, Fadhl H. Yahya, Abdullrahman M. Bawahab, Haytham M. Fayed, Ahmad Jebril M. Bosaily, Mohammed A. Bawahab

**Affiliations:** 1 Department of General Surgery, College of Medicine, King Khalid University, Abha 62529, Saudi Arabia; khassan@kku.edu.sa (K.S.A.); fsalamri@kku.edu.sa (F.S.A.A.); hahassan@kku.edu.sa (H.A.A.); dalboh@kku.edu.sa (A.D.); ac4473@kku.edu.sa (M.H.A.); malamodi@kku.edu.sa (M.A.A.); hfayed@kku.edu.sa (H.M.F.); ajmbosaily@kku.edu.sa (A.J.M.B.); mbawahab@kku.edu.sa (M.A.B.); 2 Department of General Surgery, Aseer Central Hospital, Abha 62523, Saudi Arabia; fhgy980@gmail.com; 3 College of Medicine, King Khalid University, Abha 62529, Saudi Arabia; abdullrahman.bawahab@gmail.com

**Keywords:** laparoscopic cholecystectomy (LC), sleeve gastrectomy (SG), morbid obesity, complications, efficacy, weight loss

## Abstract

**Background/Objectives:** Rapid weight loss after bariatric surgery is linked to a higher risk of symptomatic gallstone disease, which could require a cholecystectomy. This meta-analysis aimed to assess the efficacy and safety of conducting laparoscopic cholecystectomy concurrently with sleeve gastrectomy in morbid obese patients. **Methods**: Scopus, PubMed, ProQuest, Web of Science, and Google Scholar were searched for this review. Quality assessment was conducted using the Newcastle-Ottawa Scale for observational studies and ROB2 for randomized clinical trials. Eight of thirteen studies were high-quality. Pooling for BMI difference and LOS was used to measure efficacy, and the pooled proportion was utilized to assess safety through bleeding, wound infection, and leakage. Heterogeneity was explained via leave-one-out analysis and meta-regression. **Results**: The pooled standardized mean difference in BMI peri-operation was 3.26 (95% CI: 2.51–4.01, *p* < 0.01), where the age and initial BMI explained 98% of the heterogeneity through meta-regression. The pooled mean of LOS was 3.18 days (95% CI: 2.23–4.14, I^2^ = 99%, *p* < 0.001), where age explained 33.22% of the heterogeneity through meta-regression. The pooled proportion of bleeding was 0.03 (95% CI: 0.02–0.04, I^2^ = 2%, *p* = 0.42). The pooled wound infection was 0.04 (95% CI: 0.02–0.08, I^2^ = 80%, *p* < 0.001), where age accounted for 99% of the heterogeneity. The pooled biliary/gastric leakage was 0.02 (95% CI: 0.01–0.05, I^2^ = 0, *p* = 0.52). **Conclusions**: These findings indicate that the combined procedures can be executed with an acceptable safety profile. The heterogeneity in outcomes underscores the necessity of personalized patient care, standardized perioperative practices, and continuous research to enhance results and mitigate dangers.

## 1. Introduction

Obesity is an escalating global epidemic, imposing significant health-related and economic burdens. Bariatric surgery has become a recognized and successful method for attaining lasting weight loss and enhancing obesity-related comorbidities [1]. Sleeve gastrectomy (SG) is a commonly employed surgical procedure that has shown high effectiveness in facilitating weight loss and improving metabolic results. Nevertheless, fast postoperative weight reduction—especially after sleeve gastrectomy (SG)—is correlated with a heightened risk of gallstone development and symptomatic gallstone disease (GD) [2,3]. This risk is mainly due to metabolic changes after weight reduction, wherein heightened cholesterol release into the bile raises the bile-cholesterol saturation index, a crucial precursor to cholesterol gallstone formation. Consequently, numerous individuals ultimately require further cholecystectomy [4].

A meta-analysis assessing the incidence of cholecystectomy subsequent to bariatric surgery across 21 trials indicated that roughly 7.8% of patients need cholecystectomy postoperatively (95% CI, 6.0–10.1%) [5]. A recent meta-analysis by Filipe Amorim-Cruz (2023), encompassing 42 papers, estimated an 8.2% incidence of symptomatic GD post-bariatric surgeries, with variation according to the individual surgical approach employed [6]. Wuttiporn Manatsathit (2016) reported a gallstone formation rate of 47.9% in post-bariatric patients [7].

Due to the considerable prevalence of gallstone-related problems following bariatric surgery, there is an increasing interest in assessing the feasibility and safety of conducting laparoscopic cholecystectomy (LC) simultaneously with sleeve gastrectomy (SG) [8,9]. Despite being few in number, existing research indicates that this combination method may diminish the probability of later gallstone-related symptoms and the necessity for reoperation. A multicenter randomized trial indicated gallstone symptom-free rates of 91.9% at 3 months, 81.1% at 6 months, and 53.2% at 12 months after SG alone, highlighting the possible advantage of prophylactic LC in reducing delayed consequences [10].

Although numerous studies demonstrate no substantial difference in length of hospital stay (LOS) between simultaneous and phased methods [11,12], the safety of integrating LC with SG continues to be contentious. Complications like hemorrhage, biliary leakage, wound infection, and sepsis—already linked to bariatric procedures—may be intensified by simultaneous surgeries. Nonetheless, numerous studies have corroborated the safety of preventive laparoscopic cholecystectomy during laparoscopic gastric bypass, with some indicating that this strategy may be applicable to sleeve gastrectomy [13]. Mürşit Dincer (2019) observed no significant disparity in complication rates between patients receiving combination laparoscopic cholecystectomy (LC) and sleeve gastrectomy (SG) compared to those undergoing SG alone, with minimal bleeding and no instances of leakage or tissue adhesions in the combined cohort [14].

This comprehensive review and meta-analysis seek to assess the efficacy and safety of conducting laparoscopic cholecystectomy concurrently with sleeve gastrectomy in individuals with severe obesity. This study aims to address a significant gap in the literature by analyzing perioperative outcomes and postoperative complications, thereby offering information to guide therapeutic decision-making. Comprehending the dangers and advantages of the combination method may facilitate enhanced surgery planning and augment long-term outcomes for bariatric patients.

## 2. Materials and Methods

### 2.1. Study Measures

#### 2.1.1. Primary Outcomes

The primary outcome of this study was the pooled standardized mean difference (SMD) of the body mass index (BMI).

#### 2.1.2. Secondary Outcome

The secondary outcome was the pooled proportions of multiple complications associated with the concomitant laparoscopic cholecystectomy and sleeve gastrectomy.

### 2.2. Inclusion and Exclusion Criteria

Studies qualified for inclusion if they assessed the efficacy or safety of conducting LC simultaneously with SG, irrespective of publication date, language, or study design. Excluded studies comprised those lacking original research data, such as conference abstracts, posters, editorials, reviews, case reports, case series, and books. Studies that did not evaluate the results of concurrent laparoscopic cholecystectomy with sleeve gastrectomy were also eliminated. Studies devoid of quantitative outcome measures—such as alterations in BMI, cholesterol levels, or surgical complications—were excluded from analysis. To maintain methodological integrity, studies that duplicated the same patient population were eliminated. Only peer-reviewed papers with human participants were included; animal studies and in vitro research were omitted. Furthermore, studies that examined only LC or SG without assessing their combined impact were deemed ineligible. Studies pertaining to other bariatric operations (e.g., Roux-en-Y gastric bypass, adjustable gastric banding) were omitted unless they featured direct comparisons with simultaneous LC with SG.

### 2.3. Search Strategy

**Figure 1:**
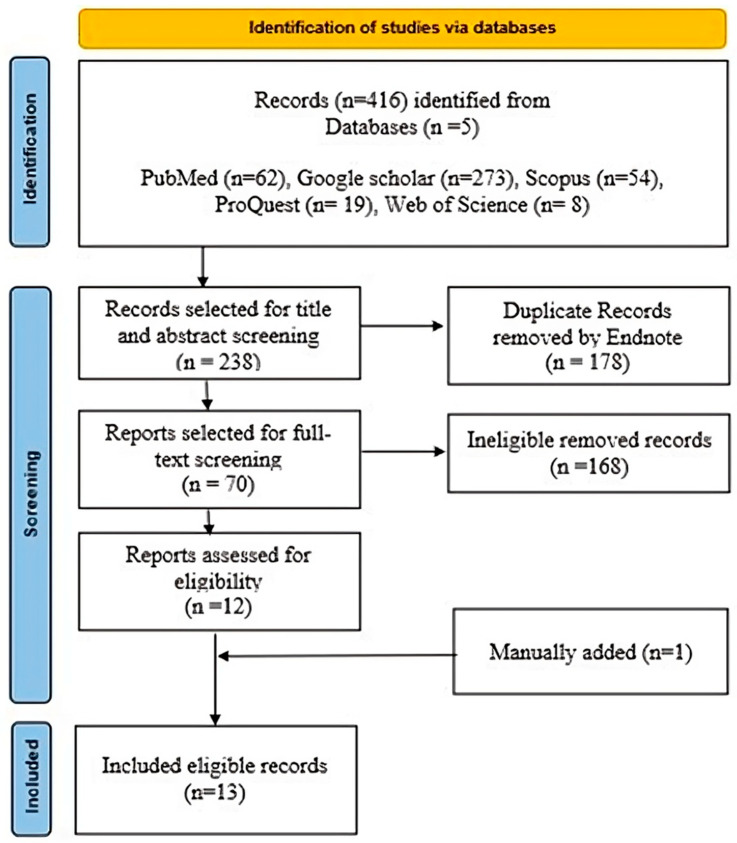
PRISMA flowchart of the included studies.

This meta-analysis adhered to the 2022 Cochrane Handbook for Systematic Reviews of Interventions and followed the Preferred Reporting Items for Systematic Reviews and Meta-Analyses (PRISMA) checklist [15,16] (Figure 1). Database searches were conducted on 13 December 2024, including Scopus, PubMed, ProQuest, Web of Science, and Google Scholar. Search terms included: “laparoscopic cholecystectomy”, “gallbladder removal”, “lap chole”, “cholecystectomy”, “sleeve gastrectomy”, “vertical sleeve gastrectomy”, “gastric sleeve”, “bariatric surgery”, “weight loss surgery”, “morbid obesity”, “severe obesity”, “class III obesity”, “obese patients”, “BMI ≥ 40”, “BMI > 35”, “outcomes”, “results”, “complications”, “adverse events”, “success rate”, “effectiveness”, “efficacy”, “postoperative recovery”, “length of stay” and “operative time”. MeSH terms were applied where appropriate. Search results were imported into EndNote X9 for duplicate detection and removal. Non-duplicate articles were exported to an MS Excel sheet for title and abstract screening, followed by a full-text review to identify eligible studies.

### 2.4. Title and Abstract Screening

Screening was conducted independently by two authors (A.D. and M.A.) based on predefined eligibility criteria. Disagreements were resolved by discussion or by a third reviewer (M.B.). The inclusion criteria were determined using the PI/ECO (Population, Intervention, Comparison, Outcome) framework, ensuring the selection of studies relevant to the efficacy and safety of concomitant laparoscopic cholecystectomy and sleeve gastrectomy in morbidly obese patients.

The population of interest comprised morbidly obese patients undergoing sleeve gastrectomy, with or without laparoscopic cholecystectomy.

The intervention/exposure was concomitant LC with SG.

The comparison group included patients undergoing sleeve gastrectomy alone.

The outcomes assessed included changes in SMD in BMI, length of hospital stay (LOS) in days, and postoperative complications such as pooled proportion of bleeding, wound infection, and leakage.

The inter-reviewer agreement for screening was 0.83, ensuring consistency and reliability in study selection.

### 2.5. Full Text Screening

All citations retrieved were downloaded for screening. If the full text was unavailable, the corresponding authors were contacted via email to request the complete manuscript. Full-text screening was conducted independently by two authors per record (F.A. and H.A.) to determine eligibility. The inter-reviewer agreement for this phase was 0.85. Any conflicts were resolved by two expert reviewers (W.A. and K.A.).

### 2.6. Data Extraction and Study Selection

Two reviewers extracted the following data: author, year, country, study designs, such as cross-sectional, cohort, or interventional studies, sample sizes, gender distribution, geographic locations, and the study outcomes. Variables not reported in the included studies were recorded as ‘not available’. Only variables reported in at least 50% of the included studies were considered for comparative analysis to ensure consistency and minimize bias due to missing data.

### 2.7. Quality Assessment

Two independent reviewers assessed the quality of included studies using the RoB 2 (released 22 August 2019) for randomized controlled trials (RCTs) [17], and Newcastle-Ottawa Scale for retrospective and prospective cohort studies and case control [18]. The latter evaluated selection, comparability, and outcome domains.

### 2.8. Statistical Analysis

Statistical analyses were conducted using R software version 4.3.2 with the “meta” package. Pooled SMD in BMI and pooled proportions of complications were calculated using a random-effects model. 

#### Heterogeneity Assessment

The random effects model was employed to calculate the pooled standardized mean difference (SMD) of the body mass index (BMI). The Tau-squared and I^2^ statistics were used to evaluate the overall heterogeneity across studies. The level of heterogeneity was categorized as low (0% to 25%), moderate (26% to 75%), or substantial (76% to 100%) [19].

### 2.9. Sensitivity Analysis

A leave-one-out sensitivity analysis was conducted to examine the impact of individual studies on the pooled SMD of BMI. In this analysis, one study was removed at a time, and the pooled proportion was recalculated to assess the influence of each study on the overall results.

### 2.10. Meta-Regression

Meta-regression was applied to explore the relationship between study-level variables and the effect size (pooled SMD of BMI). This approach was used to investigate potential sources of heterogeneity and quantify their impact on pooled estimates. It allowed for the identification of key predictors of the outcomes, helping to explain the heterogeneity observed in the analysis [20].

### 2.11. Publication Bias

Publication bias was evaluated using funnel plots, Egger’s test, or trim fill test [21].

## 3. Results

Thirteen studies reported the efficacy and safety of the combined LC with SG in 3091 morbid obese patients. The studies primarily utilized retrospective cohort designs, and one RCT [10], with sample sizes varying from 16 to 2024 persons. Most studies employed well-defined inclusion and exclusion criteria, frequently omitting individuals with a history of cholecystectomy to reduce confounding variables. The quality of the studies was predominantly high, with 9 out of 14 studies receiving a rating of ≥7. The existence of three moderate-quality studies (score ≤ 6) and significant variability in sample size and study design may lead to heterogeneity and necessitate careful interpretation of aggregated results (Table 1). The initial BMI varied between around 40 and 51 kg/m^2^, indicating a sustained emphasis on morbid obesity. Most studies indicated a female majority, consistent with global trends in bariatric surgery utilization. Comorbidity profiles differed, with hypertension and type 2 diabetes as the most commonly reported illnesses, underscoring the metabolic burden in this population. The operative duration exhibited significant variability ranging between 50.1 min (Tamer A. Habeeb, 2022) [10] and 157.2 min (Halil Coşkun, 2014) [12], with some studies reporting stratified or broad time intervals exceeding 240 min (Helmy Ezzat El Gendy, 2024) [22], likely affected by surgical methodology, institutional expertise, and the incorporation of additional operations (Table 2). The decrease in BMI after one year varied significantly from 24.63 ± 1.26 kg/m^2^ (Tamer A. Habeeb, 2022) [10] to 92.7 ± 21.0 kg/m^2^ (Azmi Lale, 2021) [23], indicating disparities in initial BMI, follow-up duration, and clinical methodologies. Bleeding rates fluctuated between 1.4% and 22.2%, and wound infection rates varied from 1.6% to 11%, reflecting procedural and institutional discrepancies. Gastric or biliary leakage happened seldom, documented in just a limited number of investigations, with a maximum frequency of 4.8%. The duration of hospital stays varied significantly, ranging from 1.76 days to nearly 5 days, affected by complication rates and postoperative care procedures. This variability highlights the necessity for uniform outcome reporting and risk adjustment in subsequent analyses (Table 3).

### 3.1. The Efficacy

#### BMI Standardized Mean Difference (SMD) Peri-Operation

**Figure 2:**
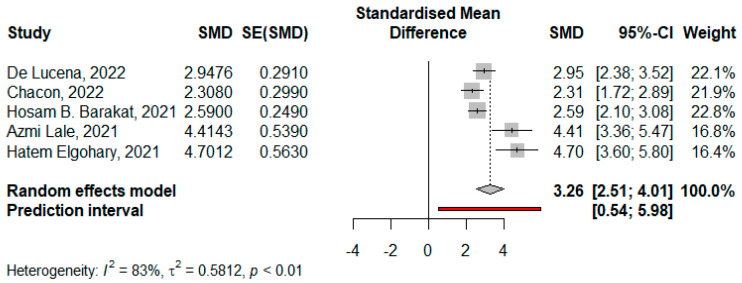
Forest plot for the mean difference in BMI peri-operation (De Lucena et al. [1], Chacon et al. [25], Barakat, H.B. et al. [11], Lale, A et al. [23], and Elgohary, H. et al. [9]).

**Figure 3:**
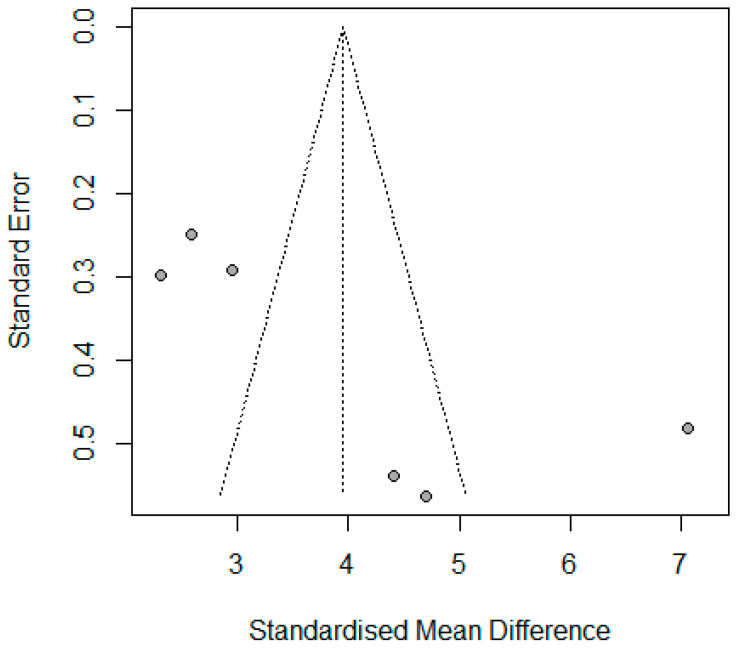
Funnel plot of mean difference in BMI reporting studies.

Six studies reported the mean change in BMI for 360 patients, where the pooled standardized mean difference (SMD) was 3.96 (95% CI: 2.54–5.39, I^2^ = 94.5%, *p* < 0.001) (Figure S1). The substantial heterogeneity decreased to I^2^ = 83% for five studies with SMD 3.26 (95% CI: 2.51–4.01, *p* < 0.01) by using leave one out analysis, which identified Tamer A. Habeeb, 2022 [10] as an outlier (Figure S2). Meta-regression was applied and revealed that age and initial BMI explained 98% of the heterogeneity, where age significantly decreased the SMD in BMI −0.336 (95% CI: −0.480–−0.192, *p* < 0.001) and initial BMI non-significantly increased SMD in BMI 0.099 (95% CI: −0.011–0.210, *p* = 0.077), tau^2^ = 1.6 (SE = 0.396). Publication bias was identified for these studies by asymmetrical funnel plot and significant Egger’s Test (*p* < 0.001) (Figure 2 and Figure 3).

### 3.2. LOS Post the Concomitant Operations

**Figure 4:**
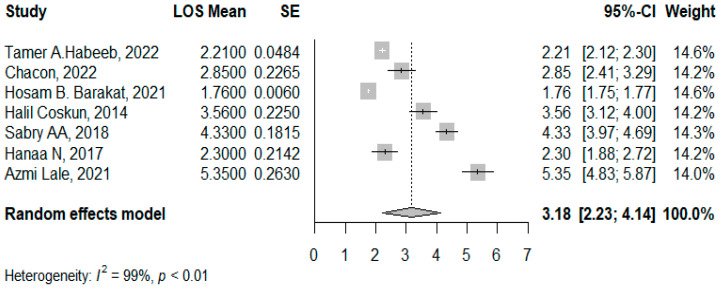
Forest plot of LOS means post the concomitant operations. (Habeeb, T.A. et al. [10], Chacon et al. [25], Barakat, H.B. et al. [11], Coskun, H. et al. [12], Sabry A.A. et al. [28], Hanaa, N. et al. [26], and Lale, A et al. [23]).

**Figure 5:**
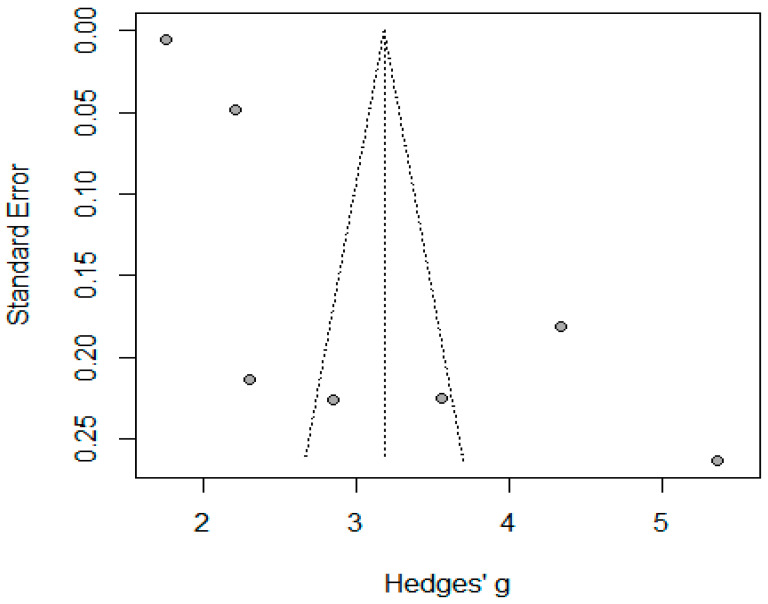
Funnel plot of the LOS reporting studies.

Seven studies reported the mean LOS following concomitant operations for 715 patients, with a pooled LOS mean of 3.18 days (95% CI: 2.23–4.14, I^2^ = 99%, *p* < 0.001). The asymmetrical funnel plot and significant Egger’s Test indicated the presence of publication bias (*p* < 0.001). Outliers, including “Hosam B. Barakat, 2021” [11] and “Azmi Lale, 2021” [23] were identified and removed, but this did not significantly reduce the high heterogeneity (I^2^ = 97%). Meta-regression analysis revealed that age accounted for 33.22% of the heterogeneity −0.219 (95% CI: −0.470–0.032, *p* = 0.087) (Figure 4 and Figure 5).

### 3.3. Complications and Safety

#### 3.3.1. Bleeding

**Figure 6:**
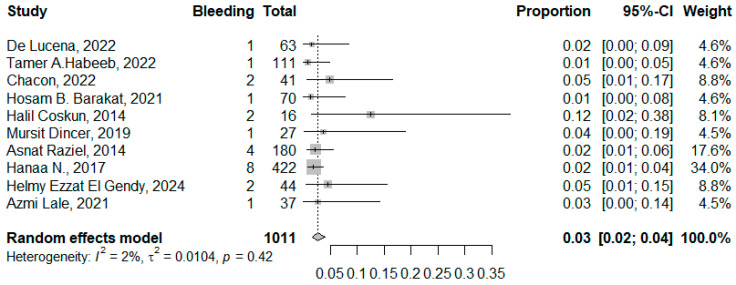
Forest plot of bleeding complications (De Lucena et al. [1], Habeeb, T.A. et al. [10], Chacon et al. [25], Barakat, H.B. et al. [11], Coskun, H. et al. [12], Dincer, M. et al. [14], Raziel, A. et al. [24], Hanaa, N. et al. [26], El Gendy, H.E. et al. [22], and Lale, A et al. [23]).

**Figure 7:**
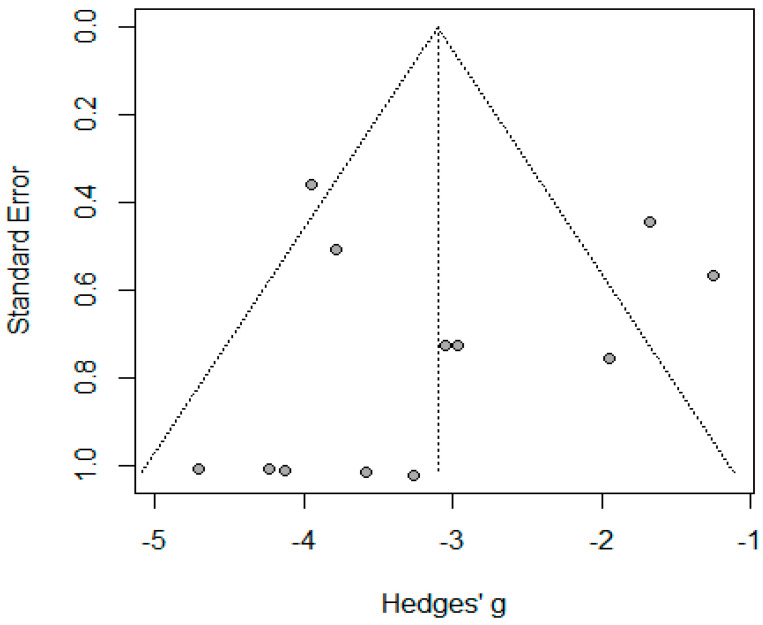
Funnel plot of studies reporting bleeding complications.

Twelve studies reported bleeding among 1067 patients, where the pooled proportion was 0.04 (95% CI: 0.02–0.08, I2 = 69%, *p* = 0.943). The symmetrical funnel plot and non-significant Egger’s test showed absence of publication bias (*p* = 0.730). The high heterogeneity was explained by identification of the outliers “Sabry AA, 2018” [28], “Hatem Elgohary, 2021” [9], where the pooled bleeding and the heterogeneity decreased to 0.03 (95% CI: 0.02–0.04, I^2^ = 2%, *p* = 0.42) (Figure 6 and Figure 7).

#### 3.3.2. Wound Infection

**Figure 8:**
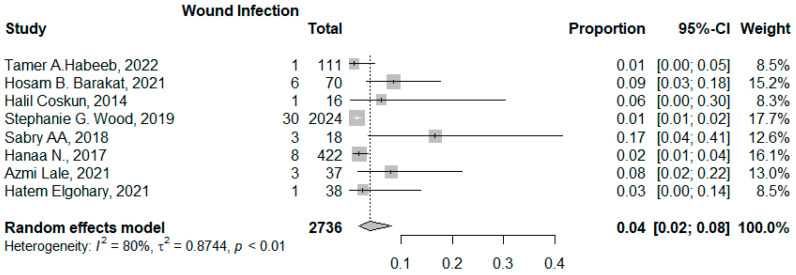
Forest plot of wound infection (Habeeb, T.A. et al. [10], Barakat, H.B. et al. [11], Coskun, H. et al. [12], Wood, S.G. et al. [8], Sabry A.A. et al. [28], Hanaa, N. et al. [26], Lale, A et al. [23], and Elgohary, H. et al. [9]).

**Figure 9:**
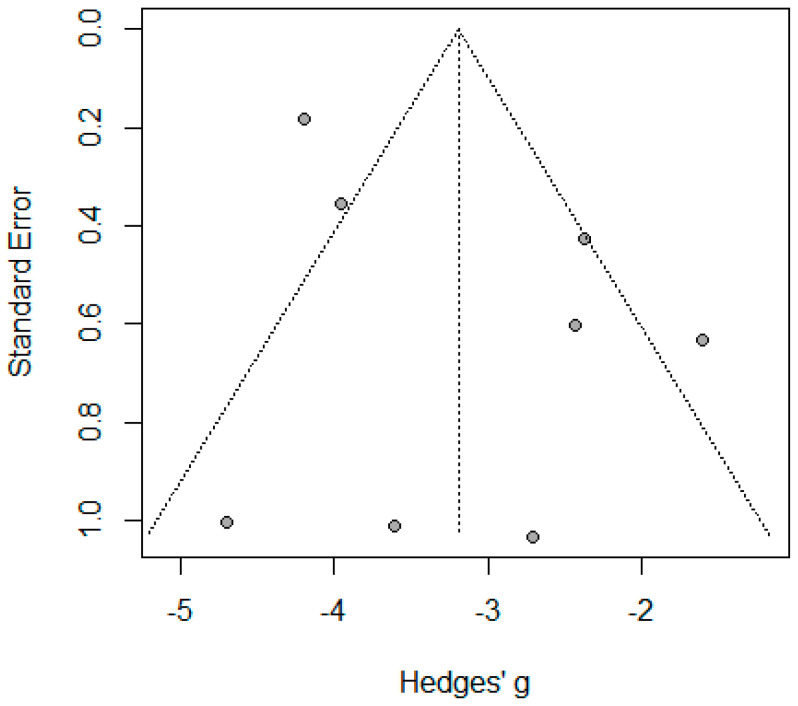
Funnel plot of studies reporting wound infection.

Eight studies reported wound infections among 2736 patients. The pooled proportion for wound infection was 0.04 (95% CI: 0.02–0.08, I^2^ = 80%, *p* < 0.001). The asymmetrical funnel plot and significant Egger’s test accounted for publication bias (*p* < 0.001). There were no outliers identified; however, meta-regression revealed that age significantly accounted for 99% of the heterogeneity among the studies −0.197 (95% CI: −0.307–0.088, *p* = 0.004) (Figure 8 and Figure 9).

#### 3.3.3. The Biliary/Gastric Leakage

**Figure 10:**
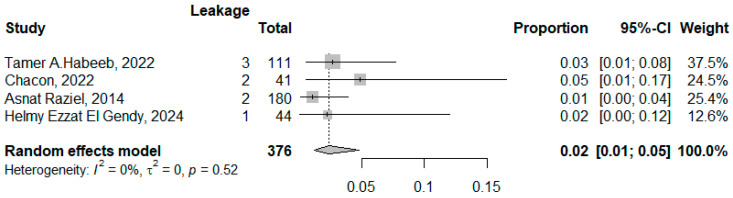
Forest plot of leakage (Habeeb, T.A. et al. [10], Chacon et al. [25], Raziel, A. et al. [24], and El Gendy, H.E. et al. [22]).

**Figure 11:**
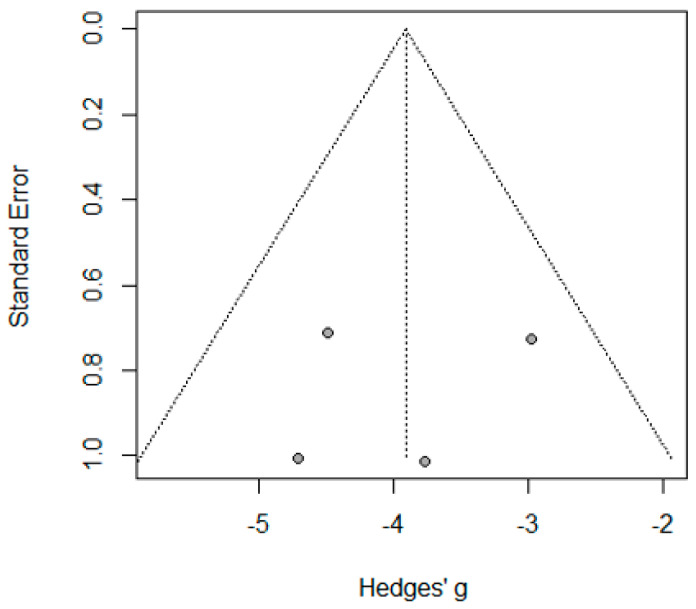
Funnel plot of studies reporting leakage.

Four studies reported for leakage among 376 patients with pooled proportion 0.02 (95% CI: 0.01–0.05, I^2^ = 0, *p* = 0.52). The symmetrical funnel plot and non-significant Egger’s Test accounted for absence of publication bias (Figure 10 and Figure 11).

## 4. Discussion

This meta-analysis assessed the effectiveness and safety of conducting simultaneous laparoscopic cholecystectomy (LC) during sleeve gastrectomy (SG) in morbidly obese individuals. The principal objective was the alteration in BMI, whereas secondary outcomes encompassed postoperative complications, including hemorrhage, wound infections, leakage, and length of stay (LOS). Thirteen studies were selected, comprising eight of high quality and five of moderate quality, with a total of 3091 patients. The analysis revealed a substantial decrease in BMI post-surgery and identified age as a primary factor contributing to variability. Publication bias and complication rates, including hemorrhage and wound infection, were also reported.

### 4.1. Effectiveness of Concurrent LC and SG

The meta-analysis indicated a substantial reduction in BMI following surgery, with a pooled standardized mean difference (SMD) of 3.96 (95% CI: 2.54–5.39; I^2^ = 94.5%, *p* < 0.001). Meta-regression indicated that age and beginning BMI collectively explained 98% of the observed variation, implying that these factors significantly affect surgery outcomes. Old-age patients with elevated baseline BMI may necessitate more rigorous preoperative treatment and exhibit greater heterogeneity in weight loss results. These findings can guide personalized patient counseling and risk stratification approaches.

A 2022 randomized controlled trial revealed a substantial disparity in excess weight loss between individuals undergoing sleeve gastrectomy (SG) alone and those undergoing simultaneous laparoscopic cholecystectomy (LC) and SG (43.27 ± 1.93 vs. 35.70 ± 5.22, *p* < 0.001) [10]. Despite only six trials presenting actual mean differences in BMI, the evidence substantiates the potential efficacy of the combined method.

The timing of cholecystectomy in bariatric patients is a critical clinical issue: whether to conduct it prophylactically during sleeve gastrectomy or postpone till symptoms manifest. This decision is frequently influenced by individual patient characteristics and institutional protocols. Although our findings indicate that a combined approach may be viable and advantageous, the absence of well-defined patient selection criteria in the included trials constrains the generalizability and robustness of these suggestions. Future research must clearly define inclusion and exclusion criteria to provide a consistent, evidence-based selection of surgical candidates.

The occurrence of gallstones in morbidly obese individuals varies from 19% to 45%, with a greater frequency noted in women of all ages [12,29,30]. Historically, laparoscopic cholecystectomy was routinely conducted during open bariatric surgeries, even in asymptomatic individuals, due to concerns regarding de novo gallstone formation resulting from rapid weight loss (30–40%), upper abdominal scarring from open surgery, and the inaccessibility of the ampulla of Vater in post-gastric bypass anatomy. Nevertheless, objections to preventative laparoscopic cholecystectomy assert that weight reduction may simplify subsequent cholecystectomy by decreasing intra-abdominal adiposity [31,32,33].

A notable constraint in the research examined is the irregular documentation of surgical methodologies and intraoperative procedures. The absence of standardization may obscure observed differences in results and impede direct comparisons. Comprehensive documenting of surgical techniques and perioperative treatment is crucial for future research to improve repeatability and interpretability.

### 4.2. Postoperative Safety and Complications

Wound infection was identified as the predominant postoperative consequence, with a pooled incidence of 4% (95% CI: 2–8%). Hanaa N. documented wound infection and sepsis rates of 1.9% and 0.5%, respectively, with no significant difference observed between SG alone and the combination treatment. Although multiple studies, including Wood et al., demonstrated no rise in mortality or serious adverse events associated with the dual operation, a substantial increase in surgical site infections was seen (*p* = 0.02) [11]. Barakat (2021) observed an elevated wound infection rate in the concurrent group (8.5% compared to 4.7%); however, the total complication rates were somewhat reduced (17.1% against 18.2%) [11]. Coşkun et al. (2014) similarly observed no significant differences in complication rates between the two groups (*p* = 0.316) [12].

However, the lack of long-term follow-up in the majority of trials limits the capacity to assess delayed or late-onset problems, such as biliary stricture, gallstone recurrence, or nutritional deficits. Standardized long-term monitoring techniques are necessary to determine the durability and safety of the combined operation over time.

### 4.3. Bleeding and Biliary/Gastric Leak

The aggregated incidence of hemorrhage and biliary/gastric leakage was comparatively low (3% and 2%, respectively), with minimal variation among studies, indicating uniformity in outcomes across cohorts. However, several studies indicated elevated bleeding rates associated with the combination surgery (*p* = 0.04) and a heightened risk of pneumonia (*p* = 0.02) [26]. Asnat Raziel (2014) noted a somewhat elevated leak rate in the combined group (1.11% vs. 0.96%) [24], whereas Chacón (2022) indicated heightened bleeding but diminished leakage, with no statistically significant difference in total morbidity (*p* = 0.288) [25]. These findings validate the viability of the combination operation while underscoring the necessity to identify procedural and patient-specific risk factors that could affect complication rates.

### 4.4. Length of Hospital Stay

The aggregated mean length of stay (LOS) was 3.18 days (95% CI: 2.23–4.14; I^2^ = 99%, *p* < 0.001), aligning with the results from Habeeb (2022), which indicated a significantly reduced LOS for the combined cohort (*p* < 0.001) [10]. Nonetheless, contradictory evidence is present. Numerous studies indicate that the use of LC may extend operating duration and length of stay due to heightened complexity [34]. Barakat (2021) indicated no statistically significant difference between groups (*p* = 0.081) [11]. The brevity of this length of stay indicates that patients receiving the combination surgery may achieve rapid recovery, perhaps resulting in decreased healthcare expenses and enhanced patient satisfaction. Conversely, it has been demonstrated that ursodeoxycholic acid (UDCA) may inhibit gallstone formation during the weight-loss period following surgery [35,36,37]. As a result, routine prophylactic CC is no longer recommended in bariatric surgeries [38].

### 4.5. Strengths and Limitations

This meta-analysis included a wide range of outcomes—such as BMI change, length of stay, and complications—offering a thorough evaluation of the dual surgical method. Advanced statistical methods, including sensitivity assessment and meta-regression, effectively addressed heterogeneity. Nevertheless, numerous constraints are present. Significant disparity in BMI outcomes and length of stay may indicate disparities in surgical approaches, patient demographics, or reporting methodologies. The experience of the surgeon, which was not recorded in the included trials, represents another significant source of variability that may affect safety and outcomes, especially in combined surgeries. The dependence on predominantly observational studies, frequently single-center and retrospective in nature, diminishes external validity. Publication bias and the omission of grey literature further constrain the generalizability of the findings. Furthermore, few trials have documented long-term outcomes, complicating the evaluation of weight reduction sustainability, late complications, or the enduring advantages of prophylactic cholecystectomy. Given these constraints, the results must be regarded with circumspection. There is a critical need for high-quality, multicenter randomized controlled trials employing established surgical methods, well-defined patient selection criteria, and long-term outcome assessment to produce conclusive data.

## 5. Conclusions

At least theoretically, this study supports the safety of performing simultaneous cholecystectomy during sleeve gastrectomy. While our findings suggest that the combined treatment may be feasible and not associated with significantly elevated risk, the existing data are limited and inconsistent. Consequently, additional high-quality prospective trials are required to corroborate these findings and provide more robust clinical evidence for identifying the most suitable candidates for this combined operation.

Future trials must prioritize the standardization of surgical techniques, furnish comprehensive documentation of inclusion and exclusion criteria, and evaluate long-term results to enhance clinical decision-making for prophylactic cholecystectomy following bariatric surgery.

## Figures and Tables

**Table 1:** Characteristics of studies included in the systematic review.

Study	Design	Sample Size	Inclusion and Exclusion Criteria	Study Quality Score
Asnat Raziel, 2014 [24] Israel	Retrospective cohort	180	Patients who were eligible for SG were included Patients with a previous cholecystectomy were excluded	5 (Moderate)
Azmi Lale, 2021 [23] Turkey	Retrospective cohort	37	Morbid obese patients undergoing LSG between January 2014 and January 2018 were included Patients who underwent previous cholecystectomy or weight reduction surgery before primary LSG were excluded	8 (High)
Chacón, 2022 [25] Spain	Retrospective cohort study	41	Patients who were eligible for SG were included Patients with a cholecystectomy prior to bariatric surgery or previous bariatric procedure were excluded.	8 (High)
de Lucena, 2022 [1] Brazil	Retrospective cohort	63	Only patients who underwent vertical sleeve gastrectomy between 2002 and 2017 were included, in addition to requiring a minimum 2-year postoperative follow-up. Patients with incomplete medical records or who were not contacted were excluded. In addition, patients in the unexposed group who had previously undergone cholecystectomy were also excluded	8 (High)
Halil Coşkun, 2014 [12] Turkey	Case-control	16	Patients who were eligible for SG were included Patients with a previous cholecystectomy were excluded	7 (High)
Hanaa N.i, 2017 [26] American database	Retrospective cohort	422	BMI < 35 kg/m^2^ were excluded from the analysis	8 (High)
Hatem Elgohary, 2021 [9] Egypt	Prospective cohort	38	BMI ≥ 35 kg/m^2^ with one or more obesity-related comorbidities and a BMI > 40 kg/m^2^ without coexisting comorbid conditions and to whom BS would not pose an excessive risk were included.	7 (High)
Helmy Ezzat El Gendy, 2024 [22] Egypt	Retrospective cohort	44	Morbid obesity and cholelithiasis eligible for surgery and underwent concomitant laparoscopic cholecystectomy and sleeve gastrectomy. Exclusion criteria: ASA III and IV patients and patients who underwent previous bariatric surgery or previous upper abdominal surgery.	6 (Moderate)
Hosam B. Barakat, 2021 [27] Egypt	Prospective cohort	70	Patients who had a previous cholecystectomy or needed additional port insertion were excluded	6 (Moderate)
Mürşit Dincer, 2019 [14] Turkey	Retrospective cohort	27	Patients with previous cholecystitis, pancreatitis history, or at least one biliary colic admitted to the hospital as symptomatic gallbladder were included. Patients who had no systemic disease other than obesity and who had asymptomatic gallstones were excluded.	5 (Moderate)
Sabry AA, 2018 [28] Egypt	Prospective cohort	18	Those who had a previous cholecystectomy were excluded	5 (Moderate)
Stephanie G. Wood, 2019 [8] United States and Canada	Retrospective cohort	2024	All patients who underwent SG with or without concurrent laparoscopic LC were included	8 (High)
Tamer A. Habeeb, 2022 [10] Egypt	Multicenter randomized trial	111	Infantile obesity, cholecystectomy, liver cirrhosis, previous bariatric surgeries, obstructive jaundice, unfit for anesthesia, difficulty in exposing gallbladder area were all excluded.	High quality (low RoB)

**Table 2:** The patients’ demographics and the operation characteristics.

Study	Initial BMI	Follow-Up Period	Comorbidities	Operative Time (min)	Age (Mean ± SD) Median (IQR) Age Group	Gender N (%)
Asnat Raziel, 2014 [24] Israel	43.1	28 months	Previous cholelithiasis 180 (100%)		46	Female 79%
Azmi Lale, 2021 [23] Turkey	45.5 ± 5.9	17.5 ± 8.1 months	Hypertension 7 (18.9%) Diabetes mellitus 10 (27.0%) Dyslipidemia 6 (16.2%) Heart diseases 2 (5.4%) Others 8 (21.6%)	82.7 ± 19.6	38.6 ± 9.3	4 Males (5.5%) 33 Females (18.5%)
Chacón, 2022 [25] Spain	-	-	Previous cholelithiasis 12 (15.58%)	152.44 ± 46.45	45.30 ± 10.49	
de Lucena, 2022 [1] Brazil	40.82 ± 4.47	2.95 ± 2.37 months	Hypertension 34 (54.0) Diabetes mellitus 10 (15.9) Dyslipidemia 24 (38.1)	-	Age group (16–29) 12 (19.0%) (30–39) 17 (27.0%) (40–49) 21 (33.3%) (50–67) 13 (20.6%)	Male 6 (9.5%) Female 57 (90.5%)
Halil Coşkun, 2014 [12] Turkey	51.1 ± 5.6	-	-	157.2 ± 40.0	39.6 ± 10.2	Male 1 (6.25%) Female 15 (93.75%)
Hanaa N.i, 2017 [26] American database	46.5 ± 7.6	30-day	Hypertension 202 (47.9) Diabetes mellitus 95 (22.5%) Dyspnea 56 (13.2%) Current smoker 38 (9%) Others 24 (5.6%)	128.2 ± 53.9	45.2 ± 11.1	Male 80 (19%) Females 342 (81%)
Hatem Elgohary, 2021 [9] Egypt	48.21 ± 8.57	12 months	Hypertension 7 (18.4%) Diabetes mellitus 4 (10.5%) Dyslipidemia 11 (28.9%)	84.19 ± 19.62	37.65 ± 10.15	Male 7 (18.4%) Female 31 (81.6%)
Helmy Ezzat el Gendy, 2024 [22] Egypt	50.10 ± 9.91	6 months	-	>60 to 120 (59.1%) >120–180 (29.5%) ≤60 min (4.5%) >180 to 240 (4.5%) >240 to 300 (2.3%)	36.48 ± 10.62	Male 3 (6.8%) Female 41 (93.2%)
Hosam B. Barakat, 2021 [11] Egypt	46.2 ± 9.95	2 years	Hypertension 8 (11.4%) Diabetes mellitus 6 (8.6%) Dyslipidemia 33 (25.7%) Previous cholelithiasis 70 (100%) Obstructive sleep apnea 2 (2.8%) Others 17 (24.3%)	76.82 ± 17.22 min	42.44 ± 10.32	Female 61 (87%) Male 9 (13%)
Mürşit Dincer, 2019 [14] Turkey	42.9 (40.8–47.5)	-	Hypertension 6 (22.22%) Diabetes mellitus 15 (55.56%) GERD 3 (11.11%) Obstructive sleep apnea 1 (3.7%) Others 6 (22.22%)	65.7 ± 8.5	40.7 ± 8.2	Male 5 (18.5%) Female 22 (81.5%)
Sabry AA, 2018 [28] Egypt	43.42 ± 2.57	30 days	-	151.56 ± 21.11	32.72 ± 6.25	Male 3 (16.7%) Females 15 (83.3%)
Stephanie G. Wood, 2019 [8] United States and Canada	44.9 (7.9)	30 days	Hypertension 978 (48%) Diabetes mellitus 422 (21%) Dyslipidemia 419 (21%) GERD 602 (30%) Obstructive sleep apnea 658 (32%) Others 428 (21.14%)	103.7 (46.2)	45.2 (12)	Females 1710 (84.5%) males 314 (15.5%)
Tamer A. Habeeb, 2022 [10] Egypt	42.7 ± 2.92	2 years	Hypertension 17 (15%) Diabetes mellitus 30 (27%)	50.13 ± 1.99	Age group (18–35) 34 (30.5%) (36–45) 69 (62%) >45 8 (7.5%)	Male 21 (19%) Female 90 (81%)

**Table 3:** The study outcomes.

Study	BMI Difference (Peri-Operation After One Year of the Operation)	Bleeding	Wound Infection	Leakage (Biliary/Gastric)	Other Complications	Length of Stay (Days)
Asnat raziel, 2014 [24] Israel	-	4 (2.22%)	-	2 (1.11%)	Incisional hernia 4 (2.2%) Others 3 (1.6%)	2 days
Azmi lale, 2021 [23] Turkey	92.7 ± 21.0	1 (2.7%)	3 (8.1%)	1 (1.6%)	Blood transfusion 1 (2.7%) Portal venous embolism: 2 (5.4%) Others 2 (5.4%)	5.35 ± 1.6
Chacón, 2022 [25] * Spain	58.30 ± 25.26	2 (4.8%)	-	2 (4.8%)	-	2.85 ± 1.45
de Lucena, 2022 [1] Brazil	28.15 ± 9.55	1 (1.4%)	-	-	-	-
Halil Coşkun, 2014 [12] Turkey	-	2 (12.5%)	1 (6.25%)	-	-	3.56 ± 0.9
Hanaa N.i, 2017 [26] American database	-	8 (1.9%)	8 (1.9%)	-	Venous thromboembolism 2 (0.47%) Sepsis/septic shock 2 (0.5%) Progressive renal insufficiency 24 (5.7%) Others 10 (2.3%)	2.3 ± 4.4
Hatem Elgohary, 2021 [9] Egypt	78.04 ± 16.6	6 (15.8%)	1 (2.6%)	-	Others 1 (2.6%)	-
Helmy ezzat el gendy, 2024 [22] Egypt	-	2 (4.5%)		Postoperative gastric leakage 1 (2.3%)	Intraabdominal collection 1 (2.3%) Extensive tissue adhesions 2 (2.3%) Postoperative subcutaneous collection1 (2.3%)	2
Hosam B. Barakat, 2021 [11] Egypt	62.48 ± 5.12	1 (1.4%)	6 (8.5%)	-	Incisional hernia 2 (2.8%) Intraabdominal collection 1 (1.4%) Others 2 (2.8%)	1.76 ± 0.05
Mürşit dincer, 2019 [14] Turkey	-	1 (3.7%)	-	-	Tissue adhesion 22 (81.48)	4 (3–4)
Sabry AA, 2018 Egypt	-	4 (22.2%)	2 (11%)	-	Intraabdominal collection 1 (5.5%) Gall bladder rupture 3 (16.7%) Others 13 (72.2%)	4.33 ± 0.77
Stephanie G. Wood, 2019 [8] United States and Canada	-	-	30 (1.5%)	-	Reoperation 33 (1.6%) Blood transfusion 10 (0.5%) Others = 3518	1.9 (1.8)
Tamer A. Habeeb, 2022 [10] Egypt	24.63 ± 1.26	1 (1.6%)	1 (1.6%)	1 (1.6%)	Reoperation 2 (3.2%) Incisional hernia 2 (3.6%)	2.21 ± 0.51

* BMI difference assessed at one year or more.

## Data Availability

The data for the review may be provided by the corresponding author on reasonable request.

## Supplementary Materials

The following supporting information can be downloaded at: https://www.mdpi.com/article/10.3390/jcm14124108/s1, the search strategy, Figure S1: SMD for BMI for the six studies, Figure S2: sensitivity of SMD (leave one out sensitivity figure), and PRISMA checklist are available [39].

## Abbreviations

The following abbreviations are used in this manuscript:

LC	Laparoscopic Cholecystectomy
SG	Sleeve Gastrectomy
BMI	Body Mass Index
LOS	Length of Stay
GD	Gallstone Disease
PI/ECO	Population, Intervention, Comparison, Outcome
RCT	Randomized Controlled Trial
RoB 2	Risk of Bias Tool 2 (Cochrane tool for assessing risk of bias)
PRISMA	Preferred Reporting Items for Systematic Reviews and Meta-Analyses
MeSH	Medical Subject Headings
ASA	American Society of Anesthesiologists (classification)
SMD	Standardized Mean Difference
I^2^	I-squared Statistic (heterogeneity measure)
SE	Standard Error
UDCA	Ursodeoxycholic Acid
IFSO	International Federation for the Surgery of Obesity and Metabolic Disorders
MBSAQIP	Metabolic and Bariatric Surgery Accreditation and Quality Improvement Program
GERD	Gastroesophageal Reflux Disease
CI	Confidence Interval

## References

[1] de Lucena A.V., Cordeiro G.G., Leão L.H., Kreimer F., de Siqueira L.T., da Conti Oliveira Sousa G., de Lucena L.H., Ferraz Á.A. (2022). Cholecystectomy concomitant with bariatric surgery: Safety and metabolic effects. Obes. Surg..

[2] Alimoğulları M., Buluş H.J.S.T. (2020). Predictive factors of gallstone formation after sleeve gastrectomy: A multivariate analysis of risk factors. Surg. Today.

[3] Adams L.B., Chang C., Pope J., Kim Y., Liu P., Yates A. (2016). Randomized, prospective comparison of ursodeoxycholic acid for the prevention of gallstones after sleeve gastrectomy. Obes. Surg..

[4] Abo-Ryia M.H., Abd-Allah H.S., El-Khadrawy O.H., Moussa G.I. (2014). Predictors of gallstone formation in morbidly obese patients after bariatric surgery: A retrospective observational study. Surg. Sci..

[5] Sattaratnamai A., Samankatiwat N., Udomsawaengsup S., Ratchaburi Hospital, King Chulalongkorn Memorial Hospital (2015). Subsequent Cholecystectomy Rate After Bariatric Surgery in Morbid Obesity Patients: A Systematic Review and Meta-Analysis.

[6] Amorim-Cruz F., Santos-Sousa H., Ribeiro M., Nogueiro J., Pereira A., Resende F., Costa-Pinho A., Preto J., Lima-Da-Costa E., Sousa-Pinto B. (2023). Risk and prophylactic management of gallstone disease in bariatric surgery: A systematic review and a Bayesian meta-analysis. J. Gastrointest. Surg..

[7] Manatsathit W., Leelasinjaroen P., Al-Hamid H., Szpunar S., Hawasli A. (2016). The incidence of cholelithiasis after sleeve gastrectomy and its association with weight loss: A two-centre retrospective cohort study. Int. J. Surg..

[8] Wood S.G., Kumar S.B., Dewey E., Lin M.Y., Carter J.T. (2019). Safety of concomitant cholecystectomy with laparoscopic sleeve gastrectomy and gastric bypass: A MBSAQIP analysis. Surg. Obes. Relat. Dis..

[9] Elgohary H., El Azawy M., Elbanna M., Elhossainy H., Omar W. (2021). Concomitant versus delayed cholecystectomy in bariatric surgery. J. Obes..

[10] Habeeb T.A.A.M., Kermansaravi M., Giménez M.E., Manangi M.N., Elghadban H., Abdelsalam S.A., Metwalli A.M., Baghdadi M.A., Sarhan A.A., Moursi A.M. (2022). Sleeve gastrectomy and cholecystectomy are safe in obese patients with asymptomatic cholelithiasis. A Multicenter Randomized Trial. World J. Surg..

[11] Barakat H.B., El-Sherpiny W.Y., Ghazaly M., Elmahdy T.M. (2021). Concomitant cholecystectomy during laparoscopic sleeve gastrectomy through the same four ports: Feasibility and early results. Egypt. J. Surg..

[12] Coskun H., Hasbahceci M., Bozkurt S., Cipe G., Malya F.U., Memmi N., Karatepe O., Akcakaya A., Muslumanoglu M. (2014). Is concomitant cholecystectomy with laparoscopic sleeve gastrectomy safe. Turk. J. Gastroenterol..

[13] Iannelli A., Treacy P., Sebastianelli L., Schiavo L., Martini F. (2019). Perioperative complications of sleeve gastrectomy: Review of the literature. J. Minimal Access Surg..

[14] Dincer M., Doğan F.J.V., Techniques O.M. (2019). The effect of concomitant cholecystectomy and sleeve gastrectomy on morbidity in high-risk obese patients with symptomatic gallstones. Videosurgery Other Miniinvasive Tech..

[15] Cochrane Handbook for Systematic Reviews of Interventions. https://training.cochrane.org/handbook.

[16] PRISMA 2020 Checklist. https://www.prisma-statement.org/prisma-2020-checklist.

[17] Cochrane Methods Bias RoB 2: A Revised Cochrane Risk-of-Bias Tool for Randomized Trials. https://methods.cochrane.org/bias/resources/rob-2-revised-cochrane-risk-bias-tool-randomized-trials.

[18] Stang A. (2010). Critical evaluation of the Newcastle-Ottawa scale for the assessment of the quality of nonrandomized studies in meta-analyses. Eur. J. Epidemiol..

[19] Higgins J.P. (2008). Cochrane Handbook for Systematic Reviews of Interventions Version 5.0.1.

[20] Baker W.L., White C.M., Cappelleri J.C., Kluger J., Coleman C.I., on behalf of the Health Outcomes, Policy, and Economics (HOPE) Collaborative Group (2009). Understanding heterogeneity in meta-analysis: The role of meta-regression. Int. J. Clin. Pract..

[21] Higgins J.P.T., Thomas J., Chandler J., Cumpston M., Li T., Page M.J., Welch V.A. (2022). Cochrane Handbook for Systematic Reviews of and N. Interventions Version 6.3.

[22] El Gendy H.E., Asar H.E.H.H., Abuzeid M.M.M. (2024). Feasibility and safety of concomitant laparoscopic cholecystectomy and sleeve gastrectomy: A retrospective study. Egypt. J. Hosp. Med..

[23] Lale A., Yur M., Do Aygen E. (2021). Safety of the concomitant cholecystectomy during laparoscopic sleeve gastrectomy in patients with symptomatic gallstone: A single-center experience. Laparosc. Endosc. Surg. Sci..

[24] Raziel A., Sakran N., Szold A., Goitein D. (2015). Concomitant cholecystectomy during laparoscopic sleeve gastrectomy. Surg. Endosc..

[25] Chacón C.G.P., Vilallonga R., López Ó.G., de Gordejuela A.G.R., Beisani M., Busquet E.C., Fort J.M., Carrasco M.A. (2022). Analysis of the management of cholelithiasis in bariatric surgery patients: A single-center experience. Obes. Surg..

[26] Dakour-Aridi H.N., El-Rayess H.M., Abou-Abbass H., Abu-Gheida I., Habib R.H., Safadi B.Y. (2017). Safety of concomitant cholecystectomy at the time of laparoscopic sleeve gastrectomy: Analysis of the American College of Surgeons National Surgical Quality Improvement Program database. Surg. Obes. Relat. Dis..

[27] Barakat H., Hassan A., Elsheikh M., Abdelhamid A. (2024). Laparoscopic single anastomosis sleeve ileal bypass in the surgical management of morbid obesity: A single-centre experience. Surg. Pract..

[28] Sabry A.A., Alkarmouty A.F. (2018). Cholecystectomy during laparoscopic sleeve gastrectomy in morbidly obese patients. Int. J. Adv. Res..

[29] Shaffer E.A. (2006). Gallstone disease: Epidemiology of gallbladder stone disease. Best Pract. Res. Clin. Gastroenterol..

[30] Gatta D.R., Huidobro L., Petermann-Rocha F., Van de Wyngard V., Godoy F., Cid V., Garrido M., Cook P., Roa J.C., Vargas C. (2024). Sex disparities in gallstone disease: Insights from the MAUCO prospective population-based cohort study. BMJ Open Gastroenterol..

[31] Fobi M., Lee H., Igwe D., Felahy B., James E., Stanczyk M., Fobi N. (2002). Prophylactic cholecystectomy with gastric bypass operation: Incidence of gallbladder disease. Obes. Surg..

[32] Worni M., Guller U., Shah A., Gandhi M., Shah J., Rajgor D., Pietrobon R., Jacobs D.O., Østbye T. (2012). Cholecystectomy concomitant with laparoscopic gastric bypass: A trend analysis of the nationwide inpatient sample from 2001 to 2008. Obes. Surg..

[33] Wudel L., Wright J., Debelak J.P., Allos T.M., Shyr Y., Chapman W.C. (2002). Prevention of Gallstone Formation in Morbidly Obese Patients Undergoing Rapid Weight Loss: Results of a Randomized Controlled Pilot Study. J. Surg. Res..

[34] Chang J., Corcelles R., Boules M., Jamal M.H., Schauer P.R., Kroh M.D. (2016). Predictive factors of biliary complications after bariatric surgery. Surg. Obes. Relat. Dis..

[35] Talha A., Abdelbaki T., Farouk A., Hasouna E., Azzam E., Shehata G. (2020). Cholelithiasis after bariatric surgery, incidence, and prophylaxis: Randomized controlled trial. Surg. Endosc..

[36] Şen O., Türkçapar A.G., Yerdel M.A. (2020). Cholelithiasis After Sleeve Gastrectomy and Effectiveness of Ursodeoxycholic Acid Treatment. J. Laparoendosc. Adv. Surg. Tech..

[37] Magouliotis D.E., Tasiopoulou V.S., Svokos A.A., Svokos K.A., Chatedaki C., Sioka E., Zacharoulis D. (2017). Ursodeoxycholic Acid in the Prevention of Gallstone Formation After Bariatric Surgery: An Updated Systematic Review and Meta-analysis. Obes. Surg..

[38] Morais M., Faria G., Preto J., Costa-Maia J. (2016). Gallstones and Bariatric Surgery: To Treat or Not to Treat?. World J. Surg..

[39] Page M.J., McKenzie J.E., Bossuyt P.M., Boutron I., Hoffmann T.C., Mulrow C.D., Shamseer L., Tetzlaff J.M., Akl E.A., Brennan S.E. (2021). The PRISMA 2020 statement: An updated guideline for reporting systematic reviews. BMJ.

